# The anti-obesity effect of mulberry leaf (*Mori Folium*) extracts was increased by bioconversion with Pectinex

**DOI:** 10.1038/s41598-022-23856-9

**Published:** 2022-11-27

**Authors:** Joo-Hui Han, Hyung-Won Lee, Sang-Hyuk Jung, Chong Woon Cho, Tae Jeong Kim, Jong Seong Kang, Chang-Seon Myung

**Affiliations:** 1 Department of Pharmacology, College of Pharmacy, Chungnam National University, Daejeon, 34134 Republic of Korea; 2 College of Pharmacy, Chungnam National University, Daejeon, 34134 Republic of Korea

**Keywords:** Biological techniques, Biotechnology, Drug discovery

## Abstract

Mulberry leaf (*Mori Folium*) extract (MLE) is known to have anti-obesity effects. In this study, the enhanced effects of MLE after bioconversion treatment using Pectinex (BMLE) on obesity were explored, and the underlying mechanisms were investigated using the active components, neochlorogenic acid (5-CQA) and cryptochlorogenic acid (4-CQA), whose amounts were increased by bioconversion of MLE. Both MLE and BMLE inhibited lipid accumulation in 3T3-L1 adipocytes without cytotoxicity and suppressed the expression of CCAAT/enhancer-binding protein alpha (C/EBPα). In addition, MLE and BMLE decreased high-fat diet-induced adipose tissue mass expansion. Notably, BMLE significantly increased antiadipogenic and anti-obesity effects compared to MLE in vitro and in vivo. The active ingredients increased by bioconversion, 5-CQA and 4-CQA, inhibited the protein levels of C/EBPα and the mRNA levels of stearoyl-CoA desaturase 1 (*Scd1*). These findings provide new insights into the therapeutic possibility of using bioconversion of MLE, by which upregulation of 5-CQA and 4-CQA potently inhibits adipogenesis.

## Introduction

Obesity is a disease in which surplus energy caused by an increase in food intake is transformed into triglycerides and stored in adipose tissue, leading to weight gain^1^. Maintaining a healthy body weight is important because obesity can cause complications such as type 2 diabetes, atherosclerosis, and heart attack^2–4^. Since the incidence of obesity has been increasing recently, it is indispensable to discover effective pharmacological drugs that potently inhibit fat accumulation for treating obesity and metabolic disease.

Transcription factors and adipogenic genes function in the molecular mechanism of adipogenesis. Adipogenic transcription factors, such as CCAAT/enhancer-binding protein (C/EBP) family members and peroxisome proliferator-activated receptor gamma (PPARγ), are essential in the adipogenesis process^5^. After *Cebpb* and *Cebpd* genes are induced to be expressed quickly in the early stages of adipocyte differentiation, these proteins increase the expression of PPARγ and C/EBPα, which are important for terminal adipocyte differentiation^6,7^. In addition, C/EBPα-mediated adipogenic transcription activation, such as stearoyl-CoA desaturase 1 (*SCD1*), has been revealed^8–10^.

SCD is an endoplasmic reticulum (ER) enzyme that catalyzes biosynthesis with monounsaturated fatty acids (MUFAs), which contribute greatly to lipid synthesis^11^. SCD has isoforms of SCD1, 2, 3, 4, 5, of which SCD1 and SCD5 are known to be expressed in humans. Furthermore, SCD1 is expressed at higher levels in adult white adipose tissue than SCD5^12–14^. Several studies have shown a link between SCD1 expression and obesity based on the finding that *Scd1* gene deficiency inhibits lipid accumulation^15,16^. Therefore, the regulation of SCD1 via C/EBPα can be a treatment strategy for obesity.

Mulberry is a plant that originated from Asia, such as Korea, China, and Japan. Traditionally, all parts of plants, such as leaves, roots, and fruits, have been used for the purpose of treating obesity or diabetes and for the purpose of eating^17,18^. Recent studies have reported the effects of mulberry leaves, such as anti-obesity^19–21^, cholesterol reduction^22,23^, anti-inflammatory^24^, antioxidant^25^, blood pressure improvement^26^, thrombosis^27^, and anti-diabetes effects^28,29^.

Bioconversion is a technology that induces the production of physiologically active ingredients by modifying the chemical structure of natural products using biological methods such as microorganisms and enzyme-mediated fermentation^30^. Several studies have reported the effect of potent therapeutic effects through bioconversion. For example, bioconverted Jeju hallabong tangor (*Citrus kiyomi* × *ponkan*) by cytolase showed antioxidant and anti-inflammatory effects^31^, and barley fermented with *Lactobacillus plantarum* dy-1 showed effects in weight loss, lipid and inflammation improvement compared with natural products^32^. Moreover, the bioconversion of *Citrus unshiu* peel extract with cytolase showed an increased inhibitory effect on adipogenic activity compared with *Citrus unshiu* peel extract^33^, and fermentation of *Panax notoginseng* by lactic acid bacteria showed an increased anti-obesity effect^34^.

Pectinex used commercially in the food industry is a fungal enzyme complex derived from *Aspergillus aculeatus*^35^. Recent studies showed that enzymatic hydrolysis of tea seed extract using Pectinex newly synthesized leucoside^36^, extracts treated with Pectinex showed a higher antioxidant effect^37,38^, and pectins extracted from rapeseed cake using Pectinex showed the effect of inhibiting cancer cell growth^39^. Therefore, it is expected that mulberry leaves extracted by grafting bioconversion technology using Pectinex will increase therapeutic activity.

We previously reported that mulberry leaf extract (MLE) after bioconversion treatment using Viscozyme L was superior to unaltered MLE in controlling diabetes both at the cellular and diabetic animal model levels^40^. In this study, we will investigate whether MLE after bioconversion treatment using Pectinex (BMLE) are more effective against obesity than MLE in 3T3-L1 adipocytes and high-fat diet-induced mouse models.

## Results

### Bioconversion of MLE enhances the inhibitory effect of adipogenesis without inducing cytotoxicity in 3T3-L1 cells

**Figure 1:**
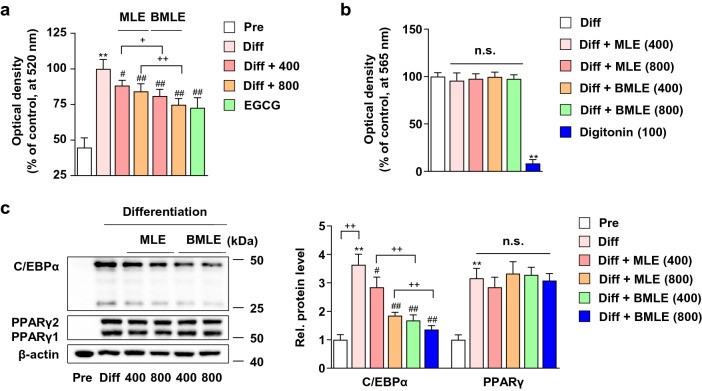
Effects of mulberry leaf extract (MLE) and bioconverted mulberry leaf extract with Pectinex (BMLE) on lipid accumulation, cytotoxicity, and adipogenic factors in 3T3-L1 cells. (**a**) Effects of MLE and BMLE on lipid accumulation in 3T3-L1 cells. MLE (400 and 800 μg/mL), BMLE (400 and 800 μg/mL) or (−)-epigallocatechin gallate (50 μM, EGCG) were treated 3T3-L1 adipocytes during differentiation periods. Oil red O staining was performed as described in the Materials and methods section. Pre: preadipocytes, Diff: 8-day differentiated cells. (**b**) Effects of MLE and BMLE on cytotoxicity in 3T3-L1 adipocytes. 3T3-L1 adipocytes were treated with MLE (400 and 800 μg/mL), BMLE (400 and 800 μg/mL) or digitonin (100 μg/mL, positive control for cytotoxicity). After 8 days of treatment, an MTT assay was performed. (**c**) Effects of MLE and BMLE on C/EBPα and PPARγ protein expression in 3T3-L1 preadipocytes and adipocytes. 3T3-L1 adipocytes were treated with MLE (400 and 800 μg/mL) and BMLE (400 and 800 μg/mL) for 8 days. ^**^P < 0.01 vs. Pre, ^#^*P* < 0.05 and ^##^*P* < 0.01 vs. Diff, ^+^*P* < 0.05 and ^++^*P* < 0.01 vs. each group, n.s.; not significant. Data are presented as the mean ± S.D.

To investigate the effect of the bioconversion of MLE on adipogenesis, we investigated the effect of lipid droplet reduction in 3T3-L1 adipocytes (8-day differentiated cells) using Oil red O staining. Compared to differentiated adipocytes, 400 and 800 μg/mL MLE dose-dependently reduced lipid droplet formation by 11.73% and 15.83%, respectively (Fig. 1a). (−)-epigallocatechin gallate (50 μM, EGCG) was used as a positive control for antiadipogenesis^41–43^. Interestingly, 400 or 800 μg/mL BMLE reduced lipid droplets by 19.00 and 25.19%, respectively, indicating that bioconversion using Pectinex significantly enhanced the inhibitory effect of lipid accumulation.

We performed an MTT assay to examine whether the inhibitory effect was due to the cytotoxicity of MLE or BMLE in 3T3-L1 adipocytes. Treatment with MLE or BMLE (400 or 800 μg/mL) for 8 days did not affect cell viability (Fig. 1b). Digitonin (100 μg/mL) was used as a positive control for cytotoxicity^44^.

To investigate which signaling pathways caused BMLE-induced adipogenesis inhibition, we performed western blots and examined the expression of C/EBPα and PPARγ proteins, major regulators of adipogenesis^45^. The expression of C/EBPα significantly decreased in the BMLE-treated groups compared to the equal dose of MLE-treated group (Fig. 1c). In contrast, MLE or BMLE treatment had no significant effect on reducing PPARγ expression. These findings suggested that bioconversion of MLE reinforced anti-adipogenesis through inhibition of C/EBPα expression.

### Bioconversion of MLE enhances suppression of adipose tissue mass in HFD-induced obese mice without inducing loss of appetite

**Figure 2:**
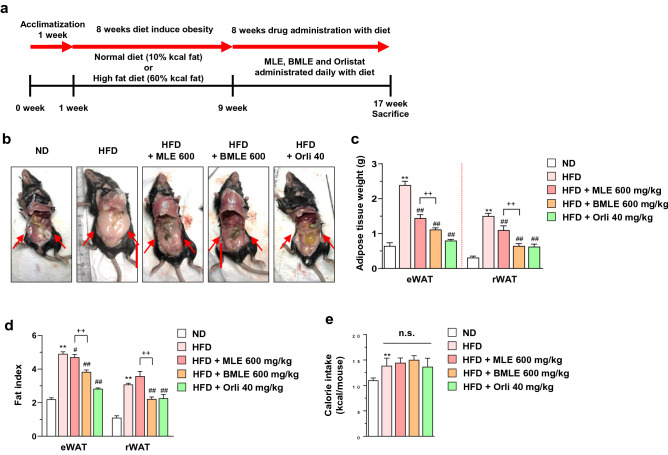
Effects of mulberry leaf extract (MLE) and bioconverted mulberry leaf extract with Pectinex (BMLE) on adipose fat mass in high-fat diet (HFD)-induced obese mice. (**a**) Schematic chart for animal study design. (**b**) Effects of MLE (600 mg/kg) and BMLE (600 mg/kg) administration on HFD-induced adipose tissue mass (n = 6 per group). Normal diet (ND)-fed mice were used as a negative control for high-fat diet-induced fat accumulation, and orlistat (40 mg/kg, orli) was used as a positive control for anti-obesity effects. (**c**) Effects of MLE and BMLE on HFD-induced epididymal adipose tissue (eWAT) and retroperitoneal white adipose tissue (rWAT) mass (n = 6 per group). (**d**) Effect of MLE and BMLE on fat index in HFD-induced mice (n = 6 per group). (**e**) Effects of MLE and BMLE on calorie intake. Fat index and calorie intake was calculated as described in the Materials and methods section. ^**^P < 0.01 vs. ND-fed mice or each group, ^#^*P* < 0.05 vs. HFD-fed mice, ^##^*P* < 0.01 vs. HFD-fed mice, ^++^*P* < 0.01 vs. each group, n.s.; not significant. Data are presented as the mean ± S.D.

To investigate the physiological effect of BMLE on adipose tissue in obesity, mice were fed ND or HFD from 5 weeks of age. After 8 weeks of obese model establishment, mice were administered MLE (600 mg/kg), BMLE (600 mg/kg) or orlistat (40 mg/kg, used as a positive control for anti-obesity^46^) for 8 weeks (Fig. 2a). The mice fed HFD had a significant increase in abdominal adipose mass compared to mice fed ND (Fig. 2b). However, increased abdominal adipose mass by HFD was inhibited by administration of MLE, BMLE or orlistat. In addition, the abdominal adipose mass of the BMLE-administered mice was severely decreased compared to that of MLE-administered mice. Furthermore, the increased eWAT and rWAT weights by HFD feeding were significantly inhibited by BMLE compared to MLE administration (Fig. 2c). As shown in Fig. 2d, the fat index increased by feeding HFD was significantly decreased in the group administered with MLE or BMLE, and the fat index decreased more in the group administered with BMLE compared with MLE. (Fig. 2d).

Since weight loss is caused by loss of appetite^47^, we examined caloric intake. MLE, BMLE and orlistat did not affect caloric intake, indicating that BMLE-mediated adipose mass inhibition was due to an antiadipogenic effect (Fig. 2e). Taken together, BMLE significantly increased the attenuation of HFD-induced adipose tissue accumulation compared to MLE without affecting caloric intake.

### Bioconversion of MLE with Pectinex increased neochlorogenic acid and cryptochlorogenic acid

**Figure 3:**
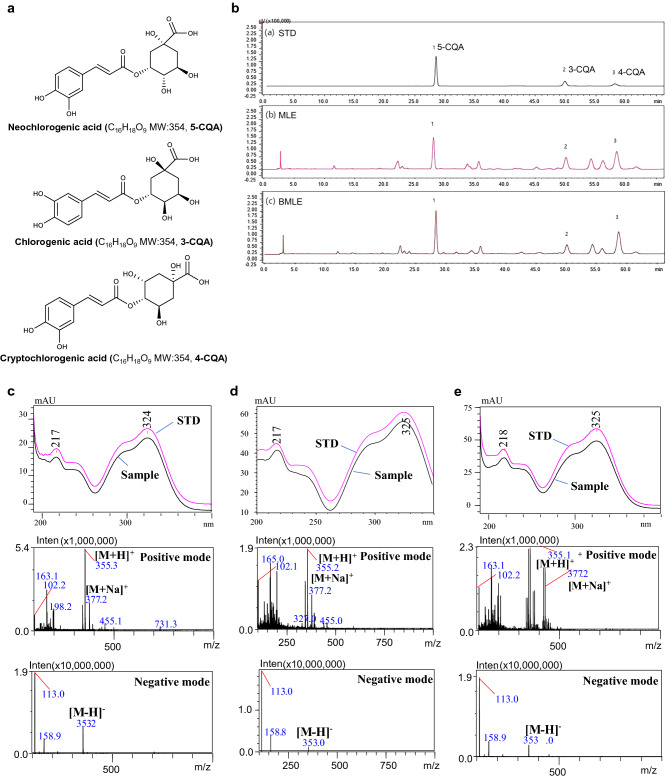
Major compound alteration by bioconversion of MLE using Pectinex. (**a**) Chemical structure of the three main compounds. (**b**) HPLC chromatogram of the standard mixture (STD), MLE and BMLE. #1, neochlorogenic acid (5-CQA); #2, chlorogenic acid (3-CQA); #3, cryptochlorogenic acid (4-CQA). UV and MS spectra of three main compounds in sample: (**c**) 5-CQA, (**d**) 3-CQA and (**e**) 4-CQA.

Bioconversion using enzymes is known to increase or decrease the amount of active components in natural products^30,48^. We compared the pattern difference of the main active components with antiadipogenic effects between MLE and BMLE by using HPLC and LC-DAD-ESI-MS (Fig. 3b–e). Among them, three compounds (#1-3) were distinguished in BMLE compared to MLE. These compounds were identified as neochlorogenic acid (#1, 5-CQA), chlorogenic acid (#2, 3-CQA) and cryptochlorogenic acid (#3, 4-CQA) from MS and UV spectra and the literature (Fig. 3a)^49–51^. These compounds, as main compounds of MLE and BMLE, were quantified with calibration curves of each compound using HPLC analysis (Fig. 3b and Supplementary Fig. S2).

The compounds 5-CQA (#1, 0.57 ± 0.03 mg/g), 3-CQA (#2, 0.09 ± 0.01 mg/g) and 4-CQA (#3, 0.06 ± 0.01 mg/g) were detected in MLE (Table 1). Additionally, 5-CQA (0.81 ± 0.02 mg/g) and 4-CQA (0.08 ± 0.01 mg/g) were detected in BMLE, respectively. Therefore, 5-CQA and 4-CQA were significantly increased by 1.42-fold and 1.33-fold, respectively, different from 3- the CQA (0.07 ± 0.01 mg/g), indicating the role of 5-CQA and 4-CQA in the bioconversion-induced enhancement of the anti-adipogenesis effect.

**Table 1:** Quantitative analysis of main compounds in MLE and BMLE (n = 6).

Compounds	Content (mg/g) ^a^		BMLE / MLE ratio
	MLE (%RSD)	BMLE (%RSD)	
5-CQA (#1)	0.57 ± 0.03 (5.26)	0.81 ± 0.02 (2.47)	1.42
3-CQA (#2)	0.09 ± 0.01 (11.11)	0.07 ± 0.01 (14.29)	0.73
4-CQA (#3)	0.06 ± 0.01(16.67)	0.08 ± 0.01 (12.50)	1.33

^a^ Data are represented as mean ± SD. *RSD* Relative standard deviation.

### Antiadipogenic effect of neochlorogenic acid and cryptochlorogenic acid

**Figure 4:**
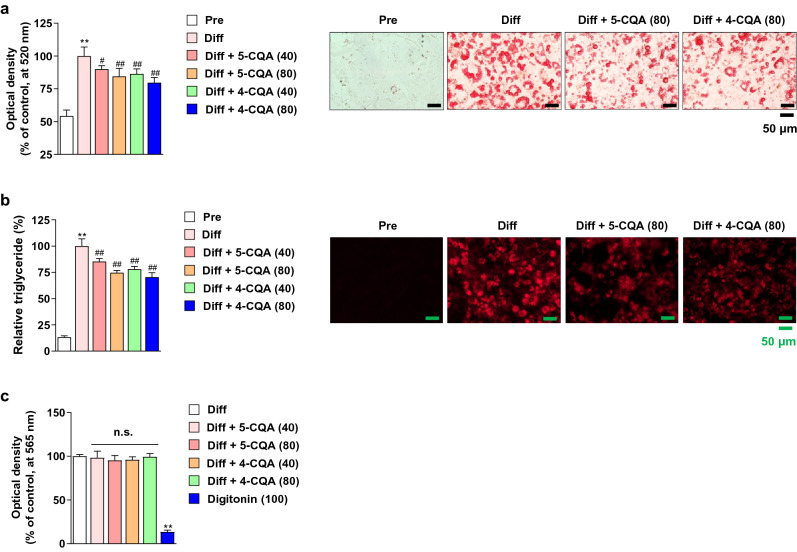
Effects of neochlorogenic acid (5-CQA) and cryptochlorogenic acid (4-CQA) on lipid accumulation, triglyceride accumulation, and cytotoxicity in 3T3-L1 cells. (**a**) Effect of 5-CQA and 4-CQA on lipid accumulation in 3T3-L1 cells. 5-CQA (40 and 80 μM) and 4-CQA (40 and 80 μM) were treated with 3T3-L1 adipocytes during differentiation periods. Oil red O staining was performed as described in the Materials and methods section. Pre: preadipocytes, Diff: 8-day differentiated cells. (**b**) Effect of 5-CQA and 4-CQA on triglyceride accumulation in 3T3-L1 cells. 5-CQA (40 and 80 μM) and 4-CQA (40 and 80 μM) were treated with 3T3-L1 adipocytes during differentiation periods. Nile red staining was performed as described in the Materials and methods section. Pre: preadipocytes, Diff: 8-day differentiated cells. (**c**) Effects of 5-CQA or 4-CQA on cytotoxicity in 3T3-L1 adipocytes. 3T3-L1 adipocytes were treated with 5-CQA (40 and 80 μM), 4-CQA (40 and 80 μM) or digitonin (100 μg/mL, positive control for cytotoxicity). After 8 days of treatment, an MTT assay was performed. ^**^P < 0.01 vs. Pre, ^#^*P* < 0.05 and ^##^*P* < 0.01 vs. Diff, n.s.; not significant. Data are presented as the mean ± S.D.

To investigate the effect of 5-CQA and 4-CQA on lipid accumulation reduction, and cytotoxicity, we performed Oil red O, Nile red assay and MTT assay in 3T3-L1 cells. The lipid droplets and triglycerides in 3T3-L1 adipocytes were significantly decreased by 40 and 80 μM 5-CQA and 4-CQA treatment without cytotoxicity (Fig. 4a-c).

### Neochlorogenic acid and cryptochlorogenic acid inhibits adipogenic factors transcription

**Figure 5:**
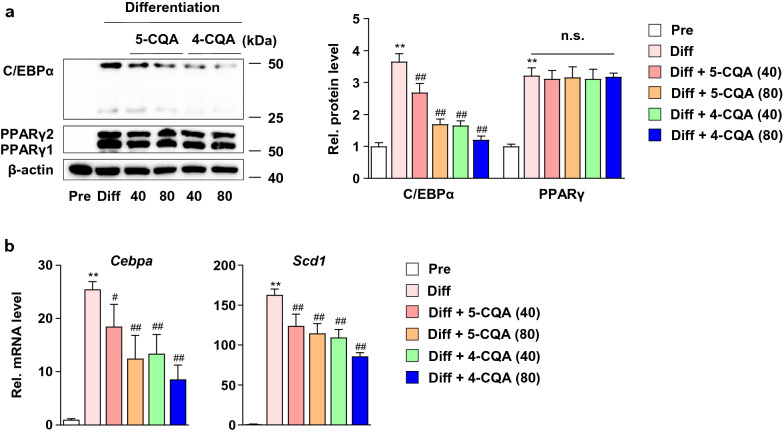
Effects of neochlorogenic acid (5-CQA) and cryptochlorogenic acid (4-CQA) on adipogenic factor expression in 3T3-L1 cells. (**a**) Effects of 5-CQA and 4-CQA on C/EBPα and PPARγ protein expression in 3T3-L1 preadipocytes and adipocytes. 3T3-L1 adipocytes were treated with 5-CQA (40 and 80 μM) and 4-CQA (40 or 80 μM) for 8 days. (**b**) Effects of 5-CQA and 4-CQA on *Cebpa* and *Scd1* mRNA expression in 3T3-L1 cells. 3T3-L1 adipocytes were treated with 5-CQA (40 and 80 μM) and 4-CQA (40 and 80 μM) for 8 days. ^**^P < 0.01 vs. Pre, ^#^*P* < 0.05 and ^##^*P* < 0.01 vs. Diff, n.s.; not significant. Data are presented as the mean ± S.D.

To investigate the effect of 5-CQA and 4-CQA on adipogenic factors, we performed Western blotting and qPCR in 3T3-L1 cells. 40 and 80 μM 5-CQA and 4-CQA treatment dose-dependently decreased C/EBPα protein levels without affecting PPARγ (Fig. 5a). Since 5-CQA and 4-CQA reduced C/EBPα protein levels, we examined the mRNA levels of *Scd1*, one of the C/EBPα downstream genes^10^, and *Cebpa*. The 5-CQA and 4-CQA treatments dose-dependently inhibited the *Cebpa* and *Scd1* mRNA levels, indicating that the C/EBPα protein was regulated by transcriptional regulation and that downregulation of C/EBPα affected adipogenic gene levels (Fig. 5b).

Taken together, we showed that the bioconversion of MLE using Pectinex significantly increased the reduction in lipid droplets in 3T3-L1 adipocytes by downregulating C/EBPα compared to MLE. In addition, BMLE administration significantly increased the reduction in adipose fat mass in HFD-induced obese mice. These effects were due to the inhibitory effect of *Cebpa* and *Scd1* expression by 5-CQA and 4-CQA, active compounds upregulated via Pectinex-mediated bioconversion.

## Discussion

Mulberry leaf (*Mori Folium*) is a well-known herb traditionally used for metabolic disease treatment^17^. In addition, there are several reports that mulberry leaf extract is effective for obesity and diabetes^19–21,28^. Bioconversion is known to increase the production of single components of natural products^30^. In the present study, we found that compared with MLE, (1) BMLE increased the reduction in lipid droplet formation and C/EBPα expression, a major regulator of adipogenesis, in 3T3-L1 adipocytes; (2) BMLE showed an enhanced anti-obesity effect by reducing the tissue mass of eWAT and rWAT in HFD-induced obese mice administered BMLE; (3) 5-CQA and 4-CQA, which are active components of MLE, were increased through bioconversion using Pectinex; and (4) the enhanced anti-adipogenesis effect of BMLE was attributed to the increased levels of 5-CQA and 4-CQA, and 5-CQA and 4-CQA inhibited adipogenesis by suppressing the *Cebpa* and *Scd1* genes.

Mulberry extract has been reported to be effective in the management of obesity^19–21^, diabetes^28^, cholesterol reduction^22,23^, anti-inflammatory^24^, antioxidant^25^, blood pressure improvement^26^, and thrombosis improvement^27^, and clinically, it has been reported to be effective in improving obesity^52^ and diabetes^53,54^. Our results showed that MLE and BMLE inhibited C/EBPα protein expression in 3T3-L1 adipocytes (Fig. 1c), and MLE or BMLE administration decreased the adipose tissue mass and fat index of eWAT and rWAT in HFD-induced obese mice (Fig. 2b‒d). According to previous results that deficiency of C/EBPα inhibits WAT fat storage^55^, modulation of C/EBPα by MLE and BMLE supported the therapeutic effect on fat accumulation and obesity. Furthermore, bioconversion of MLE using Pectinex potently reduced adipogenesis compared with MLE. Since bioactivity is enhanced by bioprocesses such as enzyme-mediated bioconversion^30^ and drug dose reduction maintaining efficacy can be possible by preventing drug safety issues, this pharmacological tool can be useful. Since Pectinex is a complex enzyme with a small amount of hemicellulose and cellulase added to the main enzyme pectinase and polygalacturonase, it can probably bioconvert plant active ingredients into a variety of compound forms as compared with single enzymes such as glycosylase or cellulase. Thus, BMLE can be an effective therapeutic method.

In the present study, we used HPLC–DAD-ESI–MS/MS to validate the major active components of BMLE. As described above, among the major active ingredients we measured, only 5-CQA and 4-CQA content increased in MLE after bioconversion treatment using Pectinex. However, it is expected that the reason why Pectinex only increased the content of 5-CQA and 4-CQA like this can be elucidated through future research. Several studies reported that 5-CQA had an anti-inflammatory effect in brain disease by inhibiting proinflammatory cytokines^56^, high potential for the development of human immunodeficiency virus-1 drug by inhibiting its reverse transcriptase activity^57^, and improved hepatic lipid accumulation and inflammation by regulating miR-34a^58^. In addition, 4-CQA was reported to have antioxidant activity^59^. However, there are not enough studies related to obesity. Our study first found that 5-CQA and 4-CQA downregulated *Cebpa* genes in 3T3-L1 adipocytes, thereby decreasing the expression of C/EBPα proteins (Fig. 5b). In addition, we found that these compounds have an antiadipogenic effect by reducing the *Scd1* genes known to induce lipid accumulation (Fig. 5b)^15^. Therefore, the increased inhibitory adipogenesis effect of BMLE was due to increased 5-CQA and 4-CQA concentrations through bioconversion using Pectinex.

## Conclusion

This study demonstrated the enhancement of the antiadipogenic and anti-obesity effects of BMLE and bioconverted MLE using Pectinex in vitro and in vivo. We showed that the major components of BMLE that were increased compared to MLE were 5-CQA and 4-CQA. In addition, 5-CQA and 4-CQA exert antiadipogenic effects by suppressing the *Cebpa* and *Scd1* genes. These findings provide new insights into the therapeutic possibility using bioconversion of MLE.

## Materials and methods

### Reagents

Dulbecco’s modified Eagle’s medium (DMEM), fetal bovine serum (FBS), bovine serum (BS), phosphate-buffered saline (PBS), penicillin/streptomycin, and trypsin-ethylenediaminetetraacetic acid (EDTA) were obtained from Gibco, Inc. (Grand Island, NY, USA). 3-Isobutyl-1-methyxanthine (IBMX), dexamethasone, insulin, 3-(4,5-dimethylthiazol-2-yl)-2,5-diphenyltetrazolium bromide (MTT), Oil red O, isopropanol, (−)‐epigallocatechin gallate (EGCG), digitonin, neochlorogenic acid (5-CQA), chlorogenic acid (3-CQA), and cryptochlorogenic acid (4-CQA) were used as reference compounds, and formic acid (HPLC grade) was obtained from Sigma–Aldrich (St. Louis, MO, USA). Anti-PPARγ, anti-C/EBPα and anti-rabbit antibodies were purchased from Cell Signaling Technology, Inc. (Beverly, MA, USA). Anti-β-actin antibody was purchased from AbFrontier (Geumcheon, Seoul, Korea). 5-CQA and 4-CQA for the in vitro study were purchased from Cayman Chemical (Ann Arbor, MI, USA). Bovine serum albumin (BSAS-AU) was purchased from Bovogen Biologicals (Keilor East, VIC, Australia). Dried mulberry leaves (*Mori Folium*) were purchased from the Naemome Dah Herb Company (Nam-gu, Ulsan, Korea). Pectinex® Ultra SP-L (3300 PGNU/g) was purchased from Novozymes (Bagsværd, Denmark). Methanol, ethyl acetate and acetonitrile of HPLC grade were obtained from Honeywell Burdick & Jackson (Muskegon, MI, USA). All other chemicals were of analytical grade.

### Preparation of MLE

The dried samples were ground using a food mixer (HMF-3080SS, Hanil Electrics, Seoul, Korea), and 2 g of sample powder was weighed, which was extracted with 20 mL of distilled water by sonication (35 kHz, 118.8 W) for 1 h at room temperature. Then, the extracted solution was centrifuged at 3500 rpm for 5 min. The supernatant was partitioned again with 20 mL of ethyl acetate for the liquid–liquid extraction (LLE) method. The LLE method was repeated three times. All water layers were evaporated at 40 °C in vacuo, and the residues were dissolved in 2 mL of methanol, which was filtered with a syringe membrane filter (PVDF, 0.22 μm) for HPLC analysis. Separately, water extracts of MLE for bioassays were filtrated with filter paper (No. 4), concentrated with a vacuum concentrator and freeze-dried to obtain a powder.

### Bioconversion of MLE

MLE solution was adjusted to pH 4.5, and Pectinex (Novozymes) was added in an amount to the 1 v/v% weight ratio of MLE solution. The mixture was then fermented at 45 °C for 15 h. Then, the mixture was incubated at 95 °C for 30 min to terminate the enzyme activity. The fermented solution was filtered with filter paper (No. 4), concentrated with a vacuum concentrator and freeze-dried to obtain a powder.

### Standard preparation and calibration curve

The stock solutions of each reference standard were prepared to 5-CQA (0.8 mg/mL), 3-CQA (0.05 mg/mL), and 4-CQA (0.6 mg/mL) using methanol and kept at 4 °C until use. To make calibration curves of the main compounds, each main compound was diluted to five different concentrations with a suitable amount of methanol as follows: 5-CQA of 1.3, 12.5, 25.0, 50.0 and 100.0 µg/mL, 3-CQA of 0.8, 1.6, 3.1, 6.3 and 12.5 µg/mL and 4-CQA acid of 0.1, 1.3, 2.5, 5.0 and 10.0 µg/mL. All standard solutions were filtered by a 0.22-μm syringe filter (PVDF) before injection into the HPLC instrument.

### Cell culture

Mouse 3T3-L1 preadipocytes were obtained from the American Type Culture Collection (ATCC CL-173™) and cultured as previously described^60^. 3T3-L1 preadipocytes were cultured in DMEM containing 10% bovine serum (BS) at 37 °C in a 5% CO_2_ atmosphere. To induce differentiation, preadipocytes were cultured in MDI [0.5 mM isobutylmethylxanthine (IBMX), 5 μM dexamethasone, 0.5 μg/mL insulin, and 10% fetal bovine serum (FBS)] and designated day 0 for differentiation. The medium was replaced every 48 h. After 2 days of differentiation, they were maintained in media containing 1 μg/mL insulin and 10% FBS. The medium containing 10% FBS was replaced every 2 days until 8 days. Cells at passages 5 to 7 were used. MLE and BMLE, 5-CQA, and 4-CQA (dissolved in distilled water) were added during the differentiation period.

### Oil red O assay

Oil red O staining was performed to observe lipid droplets as previously described^60^. Mature 3T3-L1 adipocytes were stained with Oil red O after 8 days of differentiation. Cells were fixed with 4% formaldehyde at room temperature for 1 h and washed with 60% isopropanol. Then, the fixed cells were stained with filtered Oil red O solution in 60% isopropanol. Subsequently, Oil red O was removed and washed with distilled water. Then, the stained lipid droplets were dissolved with 100% isopropanol, and the absorbance for quantification was measured at 520 nm using a microplate reader (Tecan Group Ltd., Männedorf, Switzerland). Lipid accumulation was calculated as a ratio of [(Drug treated each well O.D value)/(Control group each well O.D value)] × 100.

### MTT assay

To examine the cytotoxicity of the substance in 3T3-L1 preadipocytes, an MTT assay was performed. Cells were seeded in 96-well plates at a density of 2 × 10^4^ cells/mL and incubated at 37 °C under a humidified atmosphere of 5% CO_2_. Cells were treated with various concentrations of MLE, BMLE, 5-CQA, 4-CQA or 100 µg/mL digitonin. After 24 h, 5 mg/mL MTT reagent dissolved in serum-free DMEM was added to the wells and incubated for 2 h. After incubation, dimethyl sulfoxide was added to dissolve the crystals formed. Then, the absorbance at 565 nm was measured using a microplate reader (Tecan Group Ltd.). Cytotoxicity was calculated as a ratio of [(Drug treated each well O.D value)/(Control group each well O.D value)] × 100.

### Western blot analysis

After termination of the reaction, western blotting was performed to detect protein levels. Proteins were extracted from cells or tissues using ice-cold RIPA buffer [50 mM Tris–HCl; pH 8.0, 150 mM NaCl; 1.0% NP-40 (Sigma); 2 mM EDTA (Sigma); 5 mM NaF (Sigma); 1 mM phenylmethylsulfonyl fluoride (PMSF, Sigma); 1 mM sodium orthovanadate (Sigma); 0.5% sodium deoxycholate (Sigma) and 0.1% sodium dodecyl sulfate (SDS, Wako, Osaka, Japan)]. Denatured proteins were separated by SDS–polyacrylamide gel electrophoresis (SDS–PAGE, 10–12.5%) and transferred to polyvinylidene fluoride membranes (ATTO Corp., Tokyo, Japan). Lysates were quantified using the BCA protein Assay Kit (Pierce, Rockford, IL, USA). The membranes were blocked for 1 h with 5% BSA dissolved in TBS-T (10 mM Tris, 150 mM NaCl, and 0.1% Tween-20, pH 7.6) at 4 °C. Then, they were incubated with the appropriate primary antibodies for 16 h at 4 °C and with specific secondary antibodies for 6 h at 4 °C. Protein bands were detected using enhanced chemiluminescence reagent (ATTO Corp.), and band density was quantified with Image Lab software (Version 5.2.1, Bio–Rad, Hercules, CA, USA).

### Animals and diet

Five-week-old C57BL/6 mice (15–23 g) purchased from Orient Bio Inc. (Seongnam, Korea) were used as experimental animals in this study. Mice were acclimatized for one week in an environment controlled by temperature (22 ± 2 °C), humidity (40 ± 5%), and light (07:00 am–19:00 pm/ h intervals). For the obese mouse model, mice were fed a normal (10% kcal from fat; Research Diets, Inc., New Brunswick, NJ, USA) or high-fat diet (60% kcal fat, Research Diets, Inc.) for 8 weeks to induce an obese animal model.

After 8 weeks, five groups (n = 6/group) were randomly divided into five groups: normal diet (ND, control), high-fat diet (HFD), HFD + 600 mg/kg MLE (HFD + MLE 600), HFD + 600 mg/kg BMLE (HFD + BMLE 600), HFD + 40 mg/kg orlistat (HFD + Orli 40, positive control for anti-obesity^46^). To calculate the caloric intake, the consumed food in grams was examined and multiplied by the calories per gram of the respective type of food (ND: 3.82 kcal/g; HFD: 5.24 kcal/g). All animal experiments used in this study followed the guidelines of the Committee of Chungnam National University Laboratory Animal Ethics (CNU-00773) and The ARRIVE (Animal Research: Reporting of in vivo Experiments) guidelines^61^.

### Drug administration and dosage information

Mice were administered MLE, BMLE and orlistat dissolved in solvent orally, with a daily oral zonde needle. The daily dose of MLE (600 mg/kg) was determined by considering the doses of other studies (200–800 mg/kg)^62–65^, and the daily dose of BMLE was the same as that of MLE. In addition, the daily dose of orlistat (40 mg/kg) was determined by referring to the doses used in other experiments^46^.

### Tissue isolation

After feeding and drug administration, the mice were deeply euthanized by isoflurane, and cervical dislocation was performed. The epididymal white adipose tissue (eWAT) and retroperitoneal white adipose tissue (rWAT) were separated and weighed with an analytical electronic balance (Shimadzu Analytical Balance; Shimadzu Corporation, Kyoto, Japan). The fat index for each adipose tissue was expressed as the weight (g) of the adipose tissue per 100 g of the body weight of the individual.

### Identification of main compounds in MLE and BMLE

To identify the major components of mulberry leaf (MLE and BMLE), high-performance liquid chromatography-photo diode array detection-electrospray ionization mass spectrometry (HPLC–DAD-ESI–MS/MS) analysis was performed using the LCMS-8040 system (Shimadzu, Kyoto, Japan)*.* The LCMS-8040 system, a triple quadruple tandem mass spectrometer, consisted of a CBM-20A communication bus module, an SPD-M20A photodiode array detector, two LC-20AD pumps, a SIL-20A autosampler, and a CTO-20A column oven in positive and negative electrospray ionization (ESI) mode. Shimadzu Labsolutions software (Ver. 5.60 SP2, Kyoto, Japan) was used as the operation program. HPLC–DAD-ESI–MS/MS analysis was performed to identify the main components in the samples using an Optimak C18 column (RSTech Co., Daejeon, Korea) with mobile phases of 0.3% formic acid in water (A) and 0.3% formic acid in methanol (B), followed by a gradient elution of 5–15% B for 0–40 min and 15–19% B for 40–130 min. The main components were monitored in the UV 190–400 nm (split 1.2 nm) range. LC–MS analysis was performed under the conditions of interface voltages of 4.5 kV and − 3.0 kV of the ESI interface. Another condition was 15 L/min of a drying gas flow rate, 3 L/min of a nebulizing gas flow rate, 250 °C of a desolation line temperature and 400 °C of a heat block temperature at 0.5 mL/min of a flow rate.

### Quantitation of HPLC analysis

To quantify the amounts of the main compounds in mulberry leaves (MLE and BMLE), HPLC analysis was performed using a Shimadzu LC-20A HPLC system (Shimadzu, Kyoto, Japan) consisting of a CBM-20A communication bus module, an SPD-20A UV/VIS detector, two LC-20AD pumps, a SIL-20A autosampler and a CTO-20A column. It was operated by Shimadzu Labsolutions software (Ver. 1.25 SP4, Kyoto, Japan). HPLC analysis was performed on an Optimapak C18 column (250 × 4.6 mm (ID), 5 μm, RSTech. Co, Daejeon, Korea) with a mobile phase of 0.3% formic acid in water (A) and 0.3% formic acid in methanol (B) under the following gradient conditions: 5% ~ 20% B in 0 ~ 80 min and 20% ~ 61% B in 80 ~ 115 min, monitored at a wavelength of UV 325 nm, column temperature of 25 °C and flow rate of 1.0 mL/min. Each standard and sample solution were injected to 10 µL using an autosampler.

### Nile red staining

Nile red staining was used to measure intracellular triglycerides levels in 3T3-L1 adipocytes. Mature 3T3-L1 adipocytes were washed in PBS and treated with AdipoRed solution (Lonza, Basel. Switzerland) for 10 min. Then, the cells were washed with PBS and absorbance was measured with a microplate reader (excitation = 485 nm, emission = 535 nm; Tecan group Ltd.). Images were taken of stained cells (Olympus IX71 microscope).

### Quantitative PCR assay

Quantitative polymerase chain reaction (qPCR) was performed to determine mRNA levels. Total RNA was isolated using TRIzol reagent (Invitrogen, Waltham, MA, USA) according to the manufacturer's instructions. Complementary DNA synthesis was performed with 1 µg of RNA using the AccuPower CycleScript RT premix kit (Bioneer, Daedeok-gu, Daejeon, Korea). Real-time PCR amplification was performed using TOPreal™ qPCR Premix (Enzynomics, Yuseong-gu, Daejeon, Korea) on a 96-well optical plate. It was detected with the CFX96 real-time detection system (Bio–Rad). Under the following conditions: 1 cycle at 95 °C for 15 min; 50 cycles of 95 °C for 15 s and 60 °C for 15 s and 72 °C for 30 s, followed by 1 cycle of 65 °C to 95 °C every 0.5 °C for 1 s. In this study, only primer pairs leading to the synthesis of single fragments of appropriate size were used. The primer sets used in this study are listed in Table 2. Relative gene expression levels were calculated using the 2 − ∆∆Ct method and normalized to *Actb* expression levels.

**Table 2:** Primers for real-time PCR.

Genes	Sense primers (5′-3′)	Antisense primers (5′-3′)
*Actb*	TCCATCATGAAGTGTGACGT	GCTCAGGAGGAGCAATGAT
*Cebpa*	GAACAGCTGAGCCGTGAACT	TAGAGATCCAGCGACCCGAA
*Scd1*	AGCTGGTGATGTTCCAGAGG	AAAGTCTCGCCCCAGCAGTA

### Statistical analysis

All data are shown as the mean ± S.D. of 4–5 independent experiments. Statistical analyses were performed using GraphPad Prism software (Version 9, San Diego, CA, USA). The normality of the data distributions was tested using the Shapiro–Wilk test. A two-sided, unpaired Student’s t-test was used to analyze the difference between two groups of data with normally distributed variables. Differences among three or more groups were tested via one-way analysis of variance (ANOVA), followed by a post hoc analysis with Bonferroni’s test the *F* value was statistically significant (*P* < 0.05) and there was no significant variance in homogeneity with the Bartlett’s test. Differences were considered statistically significant at *P* < 0.05.

### Ethics approval and consent to participate

All animal experiments were approved by the Animal Experimentation Ethics Committee of Chungnam National University (Approval Number: CNU-00773), and the animals were cared for in accordance with the dictates of the National Animal Welfare Law of Korea.

## Supplementary Information

Supplementary Information 1.

Supplementary Information 2.

## Data Availability

The datasets used and/or analyzed during the current study are available from the corresponding author on reasonable request.

## Abbreviations

BMLE: Bioconverted mulberry leaf extract
C/EBP: CCAAT/enhancer binding protein
DAD: Diode array detection
EGCG: (-)-epigallocatechin gallate
ESI-MS: Electrospray ionization mass spectrometry
eWAT: Epididymal white adipose tissue
HFD: High-fat diet
HPLC: High performance liquid chromatography
MLE: Mulberry leaf extract
LC-MS: Liquid chromatography-mass spectrometry
ND: Normal diet
PPAR: Peroxisome proliferator-activated receptor
rWAT: Retroperitoneal white adipose tissue
SCD: Stearoyl-CoA desaturase
3-CQA: Chlorogenic acid
4-CQA: Cryptochlorogenic acid
5-CQA: Neochlorogenic acid
