# Supplementary figures and images for: The anti-obesity effect of mulberry leaf (Mori Folium) extracts was increased by bioconversion with Pectinex
Source: Sci Rep. 2022 Nov 27;12:20375. doi: 10.1038/s41598-022-23856-9 (PMC9701790; doi:10.1038/s41598-022-23856-9)

**Fig. 1c**

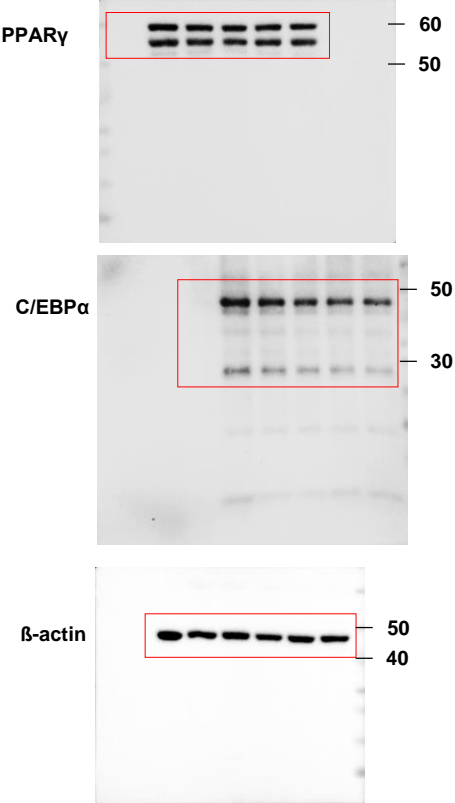

**Fig. 5a**

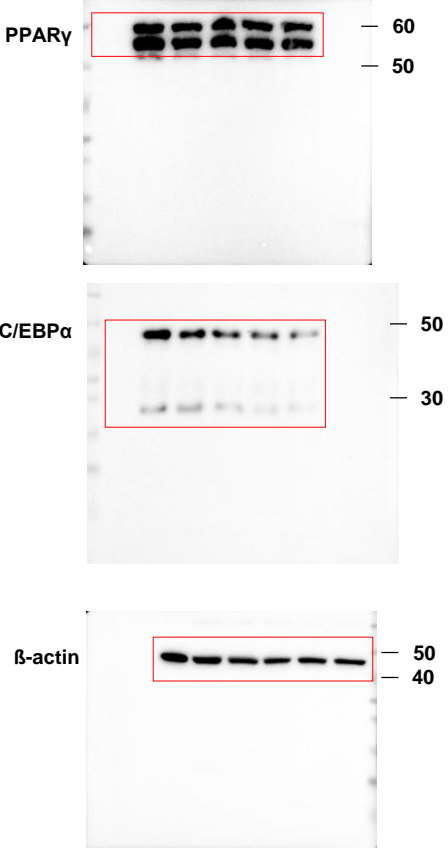

Supplement: Supplementary file 1 — Supplementary Information 1. [file 41598_2022_23856_MOESM1_ESM.pdf]
