# Supplementary material for: The anti-obesity effect of mulberry leaf (Mori Folium) extracts was increased by bioconversion with Pectinex
Source: Sci Rep. 2022 Nov 27;12:20375. doi: 10.1038/s41598-022-23856-9 (PMC9701790; doi:10.1038/s41598-022-23856-9)
Supplement: Supplementary file 2 — Supplementary Information 2. [file 41598_2022_23856_MOESM2_ESM.pdf]

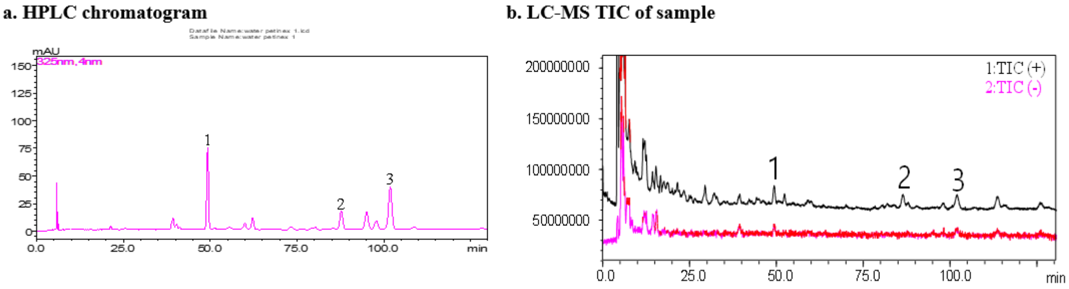

Supplementary Fig. 1 (**a**) UV and (**b**) MS spectra of three main compounds in sample: (**1**) 5-CQA, (**2**) 3-CQA and (**3**) 4-CQA

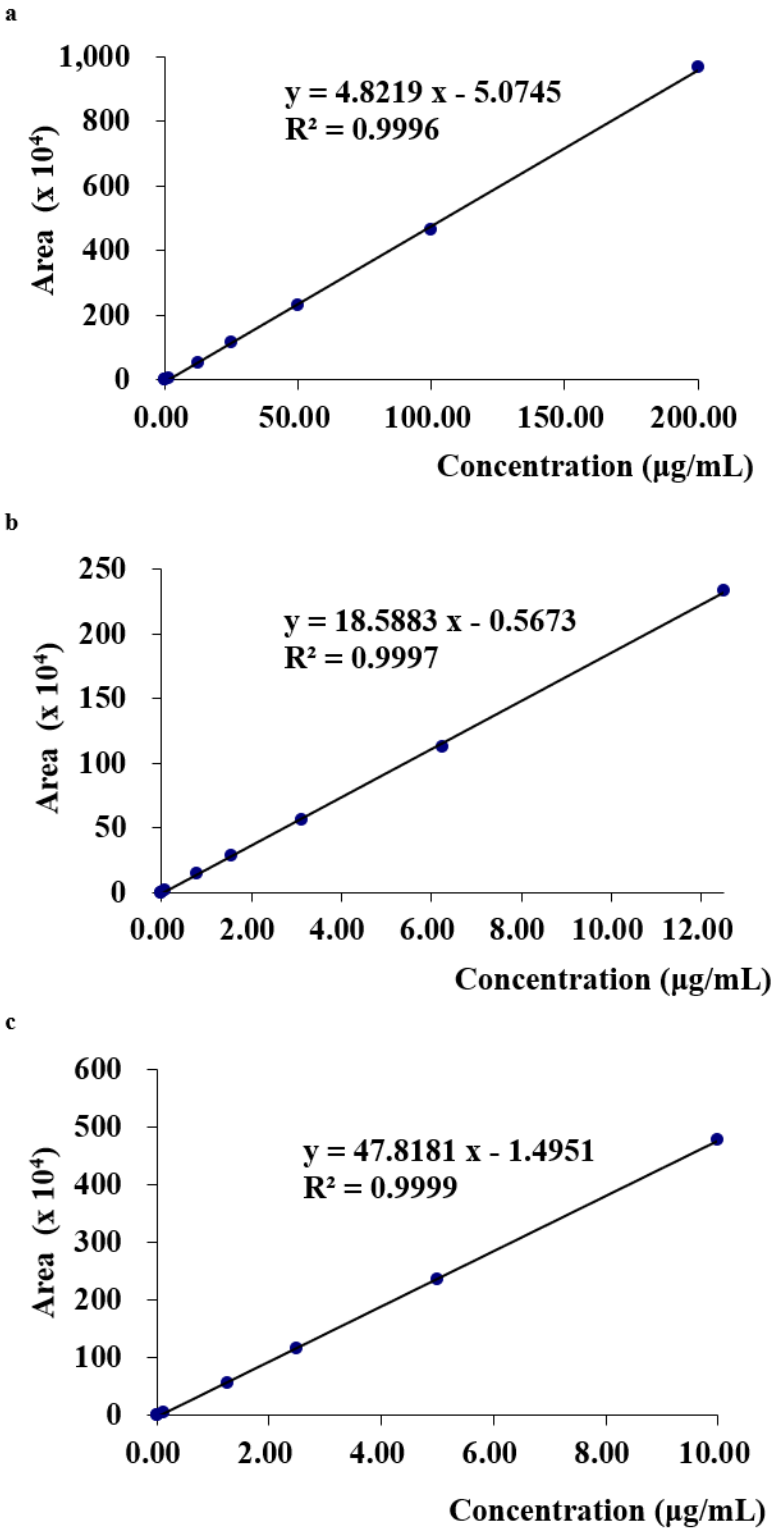

Supplementary Fig. 2 Calibration curves of three marker compounds: (**a**) 5-CQA, (**b**) 3-CQA and (**c**) 4-CQA
